# 2,4-Dinitrophenol as an Uncoupler Augments the Anthracyclines Toxicity against Prostate Cancer Cells

**DOI:** 10.3390/molecules27217227

**Published:** 2022-10-25

**Authors:** Grzegorz Adamczuk, Ewelina Humeniuk, Kamila Adamczuk, Aneta Grabarska, Jarosław Dudka

**Affiliations:** 1 Independent Medical Biology Unit, Faculty of Pharmacy, Medical University of Lublin, 20-093 Lublin, Poland; 2 Department of Biochemistry and Molecular Biology, Faculty of Medical Sciences, Medical University of Lublin, 20-090 Lublin, Poland; 3 Department of Toxicology, Faculty of Pharmacy, Medical University of Lublin, 20-090 Lublin, Poland

**Keywords:** uncoupler, 2,4-dinitrophenol, anthracyclines, doxorubicin, epirubicin, prostate cancer

## Abstract

One of the strategies for the treatment of advanced cancer diseases is targeting the energy metabolism of the cancer cells. The compound 2,4-DNP (2,4-dinitrophenol) disrupts the cell energy metabolism through the ability to decouple oxidative phosphorylation. The aim of the study was to determine the ability of 2,4-DNP to sensitize prostate cancer cells with different metabolic phenotypes to the action of known anthracyclines (doxorubicin and epirubicin). The synergistic effect of the anthracyclines and 2,4-DNP was determined using an MTT assay, apoptosis detection and a cell cycle analysis. The present of oxidative stress in cancer cells was assessed by CellROX, the level of cellular thiols and DNA oxidative damage. The study revealed that the incubation of LNCaP prostate cancer cells (oxidative phenotype) with epirubicin and doxorubicin simultaneously with 2,4-DNP showed the presence of a synergistic effect for both the cytostatics. Moreover, it contributes to the increased induction of oxidative stress, which results in a reduced level of cellular thiols and an increased number of AP sites in the DNA. The synergistic activity may consist of an inhibition of ATP synthesis and the simultaneous production of toxic amounts of ROS, destroying the mitochondria. Additionally, the sensitivity of the LNCaP cell line to the anthracyclines is relatively higher compared to the other two (PC-3, DU-145).

## 1. Introduction

Despite many therapeutic options and significant advances in medicine, cancers are one of the main causes of death worldwide, and prostate cancer is the second-leading cause of cancer death for men [1]. Due to the developing resistance to treatment during the disease progression, only the patients with localized prostate cancer may be fully cured. The knowledge gained over the decades about neoplastic diseases allows for the search for new therapeutic options for patients with advanced-stage diseases. Such a method should, on the one hand, sensitize the tumor to the applied therapy and, on the other hand, allow the use of a reduced dose of an anticancer agent in order to minimalize its side effects. One of these strategies may be targeting the cancer energy metabolism with the simultaneous use of classic anticancer agents. The effectiveness of this therapeutic option may result from the different metabolism of neoplastic cells compared to normal cells [2]. The metabolism of cancer cells is the result of many variable factors, both in the microenvironment and the increasing number of mutations accumulated during the tumor’s progression [3,4]. Moreover, the cancer cells themselves modify their microenvironment to adapt to unfavorable development conditions [5].

**Figure 1:**
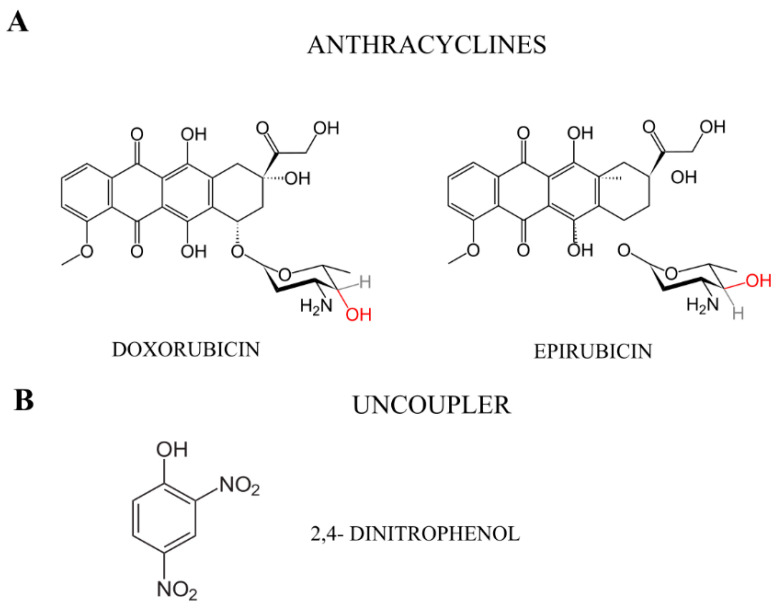
Chemical structures of (**A**) anthracyclines (doxorubicin and its derivative—epirubicin) and (**B**) uncoupler—2,4-dinitrophenol.

One example of a compound that disrupts cellular metabolism is 2,4- dinitrophenol (2,4-DNP) (Figure 1). It is known for its ability to decouple oxidative phosphorylation, which results in a reduced ATP production in cells. The changes in the mitochondrial membrane potential cause the energy to be dispersed in the form of heat, bypassing the ATP synthesis stage [6,7,8]. In addition, it also disrupts the redox balance in cancer cells [9]. In this study, 2,4-DNP was used as the disruptor of the mitochondrial function.

Regardless of recent advances in the development of targeted therapies for cancer, anthracyclines (doxorubicin and its derivate, epirubicin) are still used to treat a broad spectrum of cancers, e.g., leukemias, lymphomas, breast, stomach, uterine and ovarian [10,11]. These drugs act in the tumor cell mainly through two mechanisms: intercalation with DNA, which results in the inhibition of DNA repair by topoisomerase II and the blocking of DNA replication, as well as the production of ROS and oxidative damage to the DNA, proteins and cell membranes [10,11,12]. The resistance of cancers to chemotherapeutics is due to the high oxidative defense potential of cancer cells and the large amount of energy they produce, which is necessary for complex DNA repair processes, including DNA ligation [13,14].

Prostate cancer cell lines were used in this research due to numerous studies on their metabolic phenotype. Three lines with a potentially different metabolic phenotype were used in the study. The aim of the study was to sensitize the cells to the action of the anthracyclines, or to intensify their action through the use of the 2,4-DNP.

## 2. Result

### 2.1. Cytotoxic Analysis

**Figure 2:**
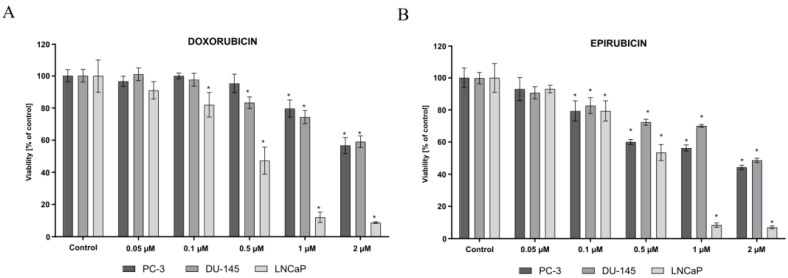
Prostate cancer cell lines (PC-3, DU-145, LNCaP) viability (% of control) based on MTT assay. The cells were treated with a wide range of (**A**) doxorubicin (0.05–2 µM) (**B**) epirubicin (0.05–2 µM) concentrations for 48 h. The values obtained from three independent experiments were presented as mean ± SD. * *p* < 0.05 vs. control.

The MTT assay revealed different sensitivities of the tested prostate cancer cell lines to the anthracyclines. Three cell lines were treated with various concentrations of anthracyclines for 48 h. As shown in Figure 2, the anthracyclines reduced the viability of the studied prostate cancer cells in a dose-dependent manner. The LNCaP cells showed greater sensitivity to the anthracyclines as compared to the other two cell lines. Only in the case of the LNCaP cell line, the IC_50_ values for both tested compounds were achieved (IC_50_ DOX—0.52 µM, IC_50_ EPI—0.54 µM). For this reason, the concentration of 0.5 µM doxorubicin and epirubicin for the LNCaP cells was chosen for the further studies, whereas, for the PC-3 and DU-145 cells, the highest concentration of the tested compounds were chosen. The doxorubicin, in the highest-tested concentration, decreased the viability of the PC-3 and DU-145 cells to about 60%, compared to the epirubicin at over 50%.

**Figure 3:**
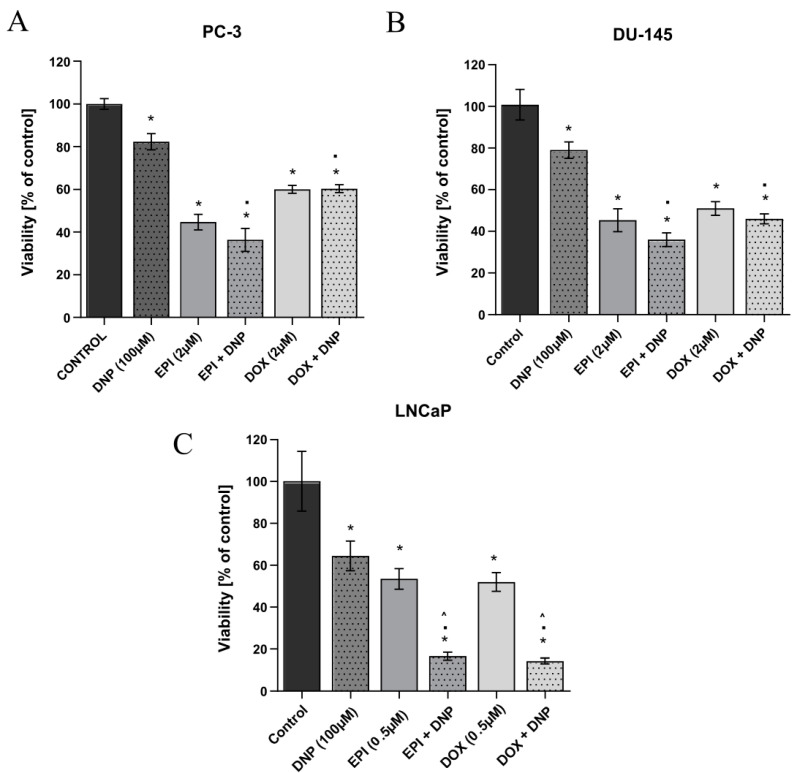
Prostate cancer cell lines (**A**) PC-3, (**B**) DU-145, (**C**) LNCaP viability (% of control) based on MTT assay. The cells were treated with 2,4-DNP and EPI (epirubicin), DOX (doxorubicin), or combined (2,4-DNP + EPI, 2,4-DNP + DOX) for 48 h. The values obtained from three independent experiments were presented as mean ± SD. * *p* < 0.05 vs. control, ▪ *p* < 0.05 vs. 2,4-DNP, ^ *p* < 0.05 vs. DOX, EPI.

In this study, 2,4-DNP is used as an uncoupler. The concentration of 100 μM of 2,4-DNP is in the range of IC_60–80_ for the tested cell lines, and, therefore, this concentration of compound was selected for the further studies. The selected concentration is below the concentration range determined in the biological fluids of patients presenting symptoms of poisoning [8]. To investigate how the decoupling of oxidative phosphorylation affects the cytotoxicity of anthracyclines, the prostate cancer cells were treated simultaneously with both the uncoupler and doxorubicin or epirubicin for 48 h (Figure 3). Only in the case of the LNCaP cell line, the simultaneous incubation of cells with the 2,4-DNP and anthracyclines caused a statistically significant decrease in the cell viability (below 20%) as compared to the viability of cells incubated with each compound alone.

**Figure 4:**
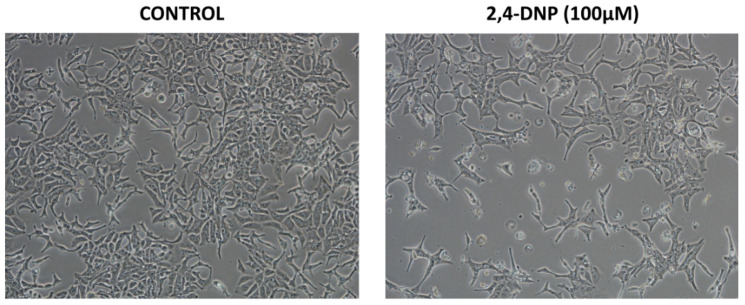

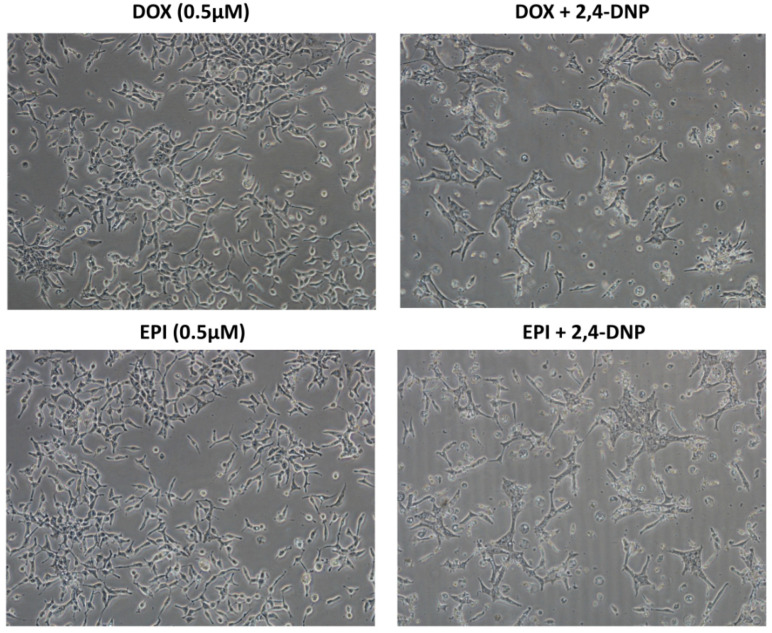
Morphological changes of LNCaP cells. The cells were treated for 48 h with 2,4-DNP (100 µM), DOX (0.5 µM) and EPI (0.5 µM) or combined (2,4-DNP + EPI, 2,4-DNP + DOX) (magnification ×100).

Therefore, only the LNCaP cells’ morphology was analyzed under a phase-contrast microscope, the Nikon Eclipse Ti (Figure 4).

The cell morphology assessment is consistent with the results obtained from the MTT assay (Figure 4). The LNCaP cells represented typical a epithelial-like morphology. The number of cells observed in the field of view was decreased after the treatment of the cells with the 2,4-DNP and anthracyclines alone. Due to the incubation with an uncoupler, the cells were comparable to the control cells, whereas the cells treated with the anthracyclines became more stellate-like in morphology, i.e., spindle-shaped.

Furthermore, the simultaneous treatment of the LNCaP cells with the 2,4-DNP and anthracyclines resulted in a visibly decreased number of cells as compared to the control. Numerous cells observed in the field of view were shrunken, dead and detached, as well as apoptotic cells being observed. The remaining cells became slightly bigger and swollen, as compared to the control cells, and lost their attachment ability.

### 2.2. Cell Cycle Analysis

**Figure 5:**
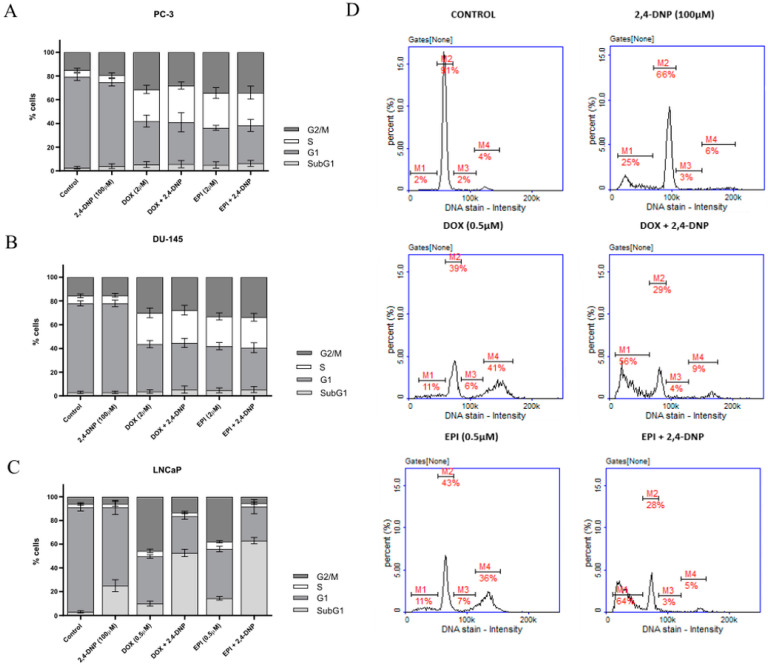
Analysis of cell cycle of (**A**) PC-3, (**B**) DU-145, (**C**) LNCaP cell lines. The cells were treated for 48 h with 2,4-DNP, DOX, and EPI or combined (2,4-DNP + EPI, 2,4-DNP + DOX). The values obtained from three independent experiments were presented as mean ± SD. (**D**) LNCaP cell cycle histograms representative of all repetitions of the experiment (M1—subG1, M2—G1, M3—S, M4—G2/M phase).

In order to investigate the mechanism of action of the tested compounds, as well as to detect their possible synergistic effect, an analysis of the cell cycle and a detection of apoptosis were performed. The cell cycle analysis showed that 2,4-DNP had no effect on changes in the cell cycle of the PC-3 and DU-145 cells. In addition, the treatment of the PC-3 and DU-145 cells with the anthracyclines, regardless of the presence of 2,4-DNP, strongly and comparably inhibited the abovementioned cells in the G2/M phase (Figure 5A,B). The cell cycle analysis of the LNCaP cells revealed that the 2,4-DNP, used alone, significantly reduced the population of cells in the G1 phase, while the peak of the subG1 phase (corresponding to the apoptotic cells) was elevated (Figure 5C,D). The anthracyclines, i.e., the DOX and EPI, presented similar patterns of histograms on the LNCaP cells—both compounds significantly inhibited cell proliferation, as reflected by an increased percentage of cells in the G2/M phase. Also, in both cases, the peak of the subG1 phase (over 5x) as compared to the control was significantly elevated. The simultaneous incubation of the LNCaP cells with the 2,4-DNP and anthracyclines contributes to a significant increase in the population of cells in the subG1 phase of the cell cycle (over 50%), while the percentage of cells in the G1 phase decreased drastically as compared to the cells incubated with each compound alone, as well as the control. Therefore, the above results indicate a synergistic effect of 2,4-DNP and anthracyclines on prostate cancer, but only on the LNCaP cell line.

### 2.3. Detection of Apoptosis

**Figure 6:**
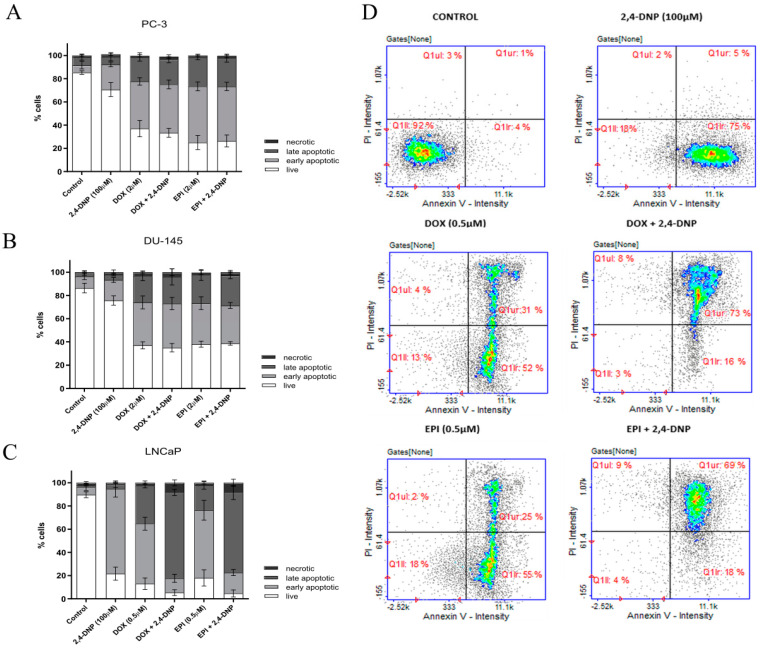
Detection of apoptosis analysis of (**A**) PC-3, (**B**) DU-145, (**C**) LNCaP cell lines. The cells were treated for 48 h with 2,4-DNP, DOX, and EPI or combined (2,4-DNP + EPI, 2,4-DNP + DOX). The values obtained from three independent experiments were presented as mean ± SD. (**D**) LNCaP cell cycle histograms representative for all repetitions of the experiment (Q1II—live, Q1Ir—early apoptotic, Q1ur—late apoptotic, and Q1uI—necrotic cells).

In the case of the PC-3 and DU-145 cell lines, the detection of apoptosis indicated that the 2,4-DNP had a small impact on the increase in the number of cells in the early phase of apoptosis (Figure 6A,B). On the contrary, the simultaneous incubation of cells with anthracyclines and 2,4-DNP significantly increased the number of cells in the early and late stages of apoptosis (with a predominance of cells in the early phase), regardless of the 2,4-DNP being used.

In contrast, an analysis of the apoptosis revealed that almost all the LNCaP cells treated with the 2,4-DNP were early apoptotic (Figure 6C,D). After the anthracyclines treatment, the cells were in the early stages of apoptosis, as well as in the late stages. In the case of the simultaneous DOX and 2,4-DNP treatment, the number of cells in the late phase of apoptosis increased. A similar pattern has been observed in the case of the EPI.

### 2.4. Detection of Oxidative Stress

**Figure 7:**
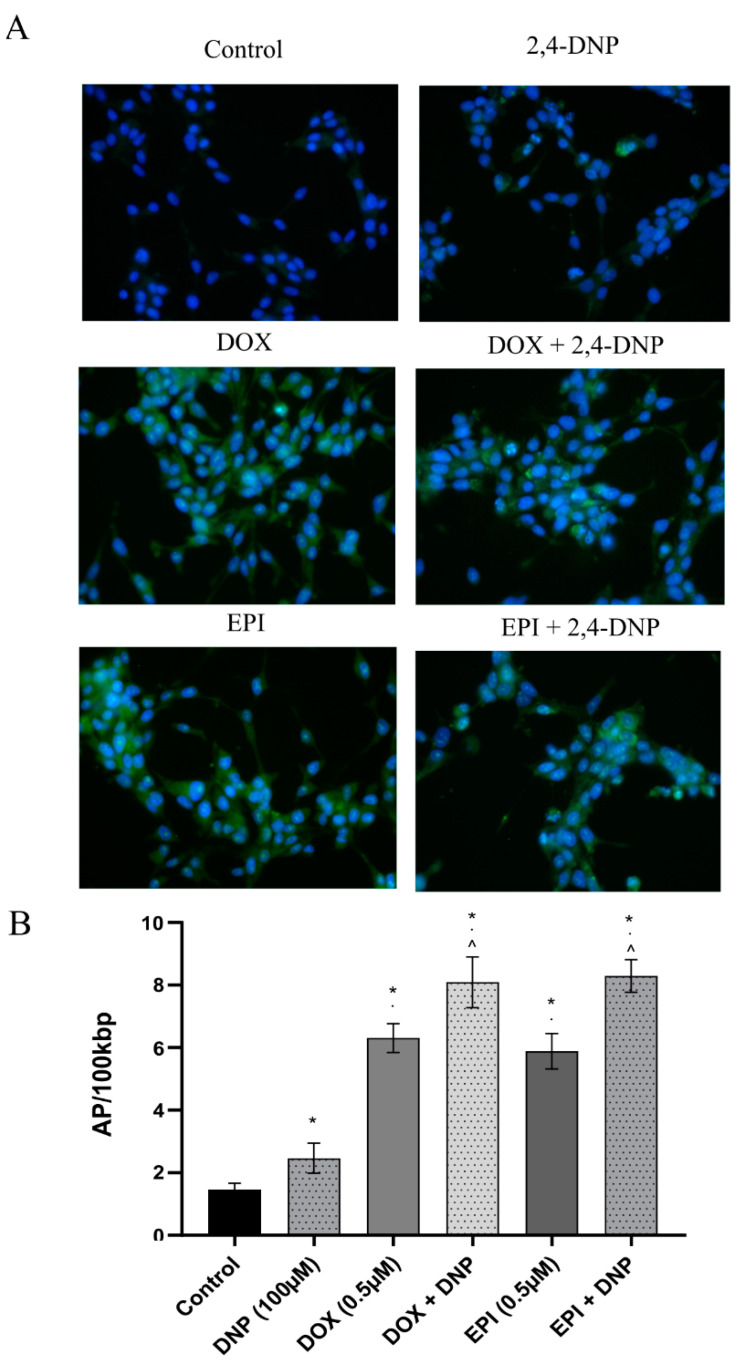
(**A**) The presence of oxidative stress in LNCaP cells. The cells were treated for 24 h with 2,4-DNP, DOX and EPI or combined (2,4-DNP + EPI, 2,4-DNP + DOX). (**B**) AP sites’ level in DNA of LNCaP cells. The cells were treated for 48 h with 2,4-DNP, DOX and EPI or combined (2,4-DNP + EPI, 2,4-DNP + DOX). The values obtained from three independent experiments were presented as mean ± SD. * *p* < 0.05 vs. control, · *p* < 0.05 vs. 2,4-DNP, ^ *p* < 0.05 vs. DOX, EPI.

CellROX Green Reagent was used to detect the ROS generation in the cells. Its bright, green fluorescence is observed after oxidation by the ROS and bonding to the DNA (in the nucleus and mitochondria). A small fluorescent signal derived from the mitochondria was observed in the cells treated with the 2,4-DNP (Figure 7A). In the case of the anthracyclines, a high signal came mainly from the nuclei. The combined treatment resulted in the generation of the oxidative stress signal in both the nuclei and mitochondria.

**Figure 8:**
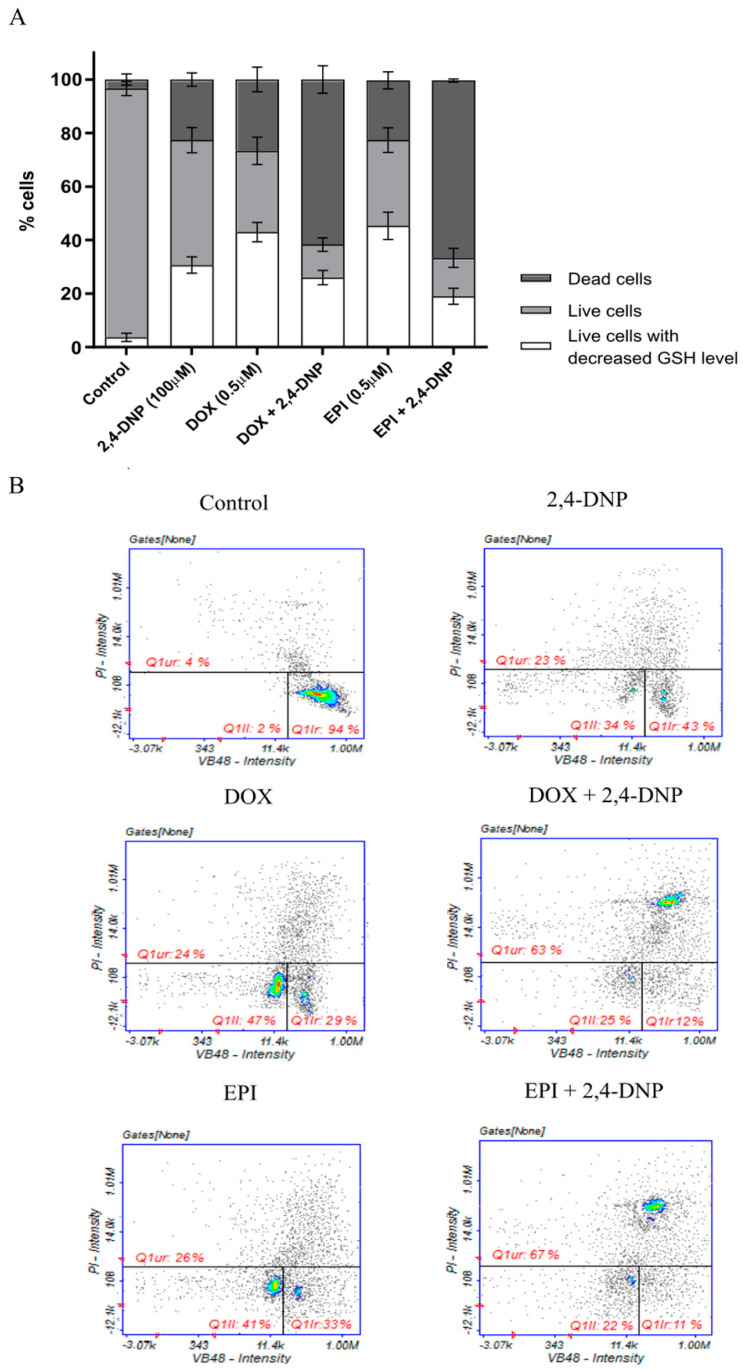
(**A**) Detection of the level of cellular thiols in LNCaP cells by image cytometry. The cells were treated for 24 h with 2,4-DNP, DOX and EPI or combined (2,4-DNP + EPI, 2,4-DNP + DOX). The values obtained from three independent experiments were presented as mean ± SD. (**B**) Histograms representative for all repetitions of the experiment (Q1II-PI negative cells with decreased GSH level, Q1Ir-healthy cells, Q1ur-dead cells).

The measurement of the cellular thiols levels was performed to determine whether the synergistic effect occurred due to disturbances in the level of intracellular glutathione (GSH) in the LNCaP cells. GSH is an essential component of the antioxidant defense system, and its dysregulation indicates a redox imbalance in the cells. Thus, the GSH level is commonly used in the measurement of the oxidative stress status. The assay revealed that the LNCaP cells treated with the tested compound alone, as well as combined, significantly decreased the GSH level as compared to the control cells (Figure 8). In addition, in the case of the LNCaP cells treated simultaneously with both an uncoupler and the anthracyclines, the percentage of the dead cells was increased as compared to the cells treated with the aforementioned compounds separately.

The oxidative DNA damage assessment was performed to determine whether oxidative stress impacted the number of AP sites. There was a significant increase in the AP sites’ number in the DNA isolated from the LNCaP cells treated with the 2,4-DNP (2.47 ± 0.48 AP sites/100,000 bp) as well as the doxorubicin (6.31 ± 0.46 AP sites/100,000 bp) and epirubicin (5.89 ± 0.57 AP sites/100,000 bp), as compared to the control culture (1.47 ± 0.2 AP sites/100,000 bp) (Figure 7B). Furthermore, after combining the 2,4-DNP with the anthracyclines, the AP sites’ accumulation in the DNA significantly increased (2,4-DNP + DOX—8.09 ± 0.81 AP sites/100,000 bp, 2,4-DNP + EPI—8.29 ± 0.52 AP sites/100,000 bp) in comparison to the anthracyclines used alone.

## 3. Discussion

In the process of selecting the appropriate therapy for an oncological patient, it is more and more frequently assumed that the neoplasm is a multiclonal disease. The multi-clonality may be responsible for the different sensitivity of individual cell clones to the therapies. Tumor cell resistance increases with the disease progression, due to the accumulation and/or emergence of further mutations in the cancer cells. A major problem is that single cancer cell clones remain after the treatment. They are responsible for the recurrence and development of metastases and are resistant to the previously applied therapy. Taking the above into consideration, the search is constantly ongoing to find new solutions to overcome the problem of therapeutic efficacy and drug resistance. Taking into account the economic aspect, it could allow a wider access to the therapy. One of the proposals of such a strategy is the combination of classical therapy, i.e., chemotherapy, with other agents of moderate toxicity. The choice of an additional factor enhancing the effectiveness of classical therapy may be supported by the knowledge about tumor biology. Multiple mutations arising during cancer transformation are not only directly related to proliferation, which is the essence of cancer development, but also to the change in the activity of many cell pathways [3,4]. One of such changes observed in most types of cancers is the altered activity of the energy acquisition pathways, which is manifested in changes in the metabolic phenotype of the cancer cell. This effect is known as the Warburg effect and is based on the activation of glycolysis, despite unlimited access to oxygen [15,16,17].

Thus, one of the strategies for developing new anticancer therapies may be to disrupt the proper functions of the mitochondria. One example of this strategy is using metformin as an anticancer drug. Some evidence suggests that metformin, currently used as an antidiabetic drug, inhibits complex I of the electron transport chain, induces apoptosis and reduces cancer cell proliferation [18,19,20]. Another possibility could be the uncoupling of oxidative phosphorylation, e.g., by 2,4-DNP. The poor functioning of the glycolytic pathway, and thus a strong dependence on mitochondrial respiration, is the basis for the assumption that the use of compounds disrupting the function of the mitochondria will result in the synergy of these compounds with classic chemotherapeutic agents. The main aim of the study was to assess whether a substance that disrupts the function of the mitochondria by OXPHOS uncoupling in a prostate cancer cell would be able to sensitize it to anthracyclines, as well as to enhance its effectiveness.

This study focused on three prostate cancer lines with a known and documented metabolic phenotype. Higgins et al. [21] found that the three classical prostate cancer cell lines (LNCaP, DU-145, PC-3) show dramatic differences in their metabolic phenotype. The LNCaP cells had a more oxidative phenotype in contrast to both the DU-145 and PC-3 cells. That is, the LNCaP cells have higher rates of oxygen consumption and lower rates of lactate production than the DU-145 and PC-3 cell lines [21].

The close dependence of the LNCaP cell line on oxidative phosphorylation is evidenced by the decrease in viability in the presence of 2,4-DNP, the largest among the tested cell lines. As already mentioned, the incubation of the LNCaP prostate cancer cells with epirubicin and/or doxorubicin simultaneously with 2,4-DNP allowed us to observe the presence of a synergistic effect for both the cytostatics. Additionally, the sensitivity of the LNCaP cell line to the anthracyclines is relatively higher compared to the other two (PC-3, DU-145). A common mechanism for anthracyclines and 2,4-DNP may be the disruption of NADH utilization. The 2,4-DNP causes the disruption of ATP formation and increases the mitochondrial requirement for NADH. On the other hand, anthracycline-associated cardiolipin is reduced by the NADH in complex II of the mitochondrial transport chain, resulting in insufficient amounts of this nucleotide to generate the appropriate amount of ATP [22]. In addition, the electron transferred from the NADH to the anthracyclines is transferred to molecular oxygen, resulting in the formation of a superoxide radical, which is a precursor of a much more toxic reactive oxygen species [23]. Thus, an impaired redox balance and an excess of ROS can cause oxidative damage to the proteins, lipids and mitochondrial DNA. GSH plays a key role in the cellular maintenance of the redox balance. Lash et al. [24], in a study conducted on the PC-3 and LNCaP lines, observed a 4.2-fold increase in the GSH concentration in PC-3 cells compared to LNCaP cells [24]. Similar results were obtained by Chaiswing et al. [25]. The research revealed that the mean concentrations of GSH and glutathione disulfide (GSSG) were significantly higher in the PC-3 cells than in the LNCaP cells [25]. In turn, Childs et al. [26] observed a lower GSH level in the LNCaP line than in the DU-145 line [26]. The results of the above studies indicate that the lower level of cellular GSH in the LNCaP cells may be the reason for the greater sensitivity of the tested line to the action of the anthracyclines, and thus may be responsible for the synergistic effect.

It can be assumed that the synergy of the cytotoxic activity may consist of the inhibition of ATP synthesis and the simultaneous production of toxic amounts of ROS, destroying the mitochondria. Mitochondrial respiration constitutes the most important source of ROS in the cell. The majority of uncouplers (including 2,4-DNP) were shown to increase the ROS levels in cancer cells and often decreased the antioxidant defense by lowering the GSH and NADPH levels [9,27]. The conducted studies confirmed that the simultaneous incubation of cells with 2,4-DNP and anthracyclines contributes to the increased induction of oxidative stress, which results in a reduced level of cellular thiols with an elevated number of necrotic cells in the LNCaP cells. In addition, a higher level of oxidative stress in the LNCaP cells was responsible for the increased number of AP sites in the DNA. It, therefore, seems highly probable that the sensitivity of the LNCaP cell line may result from its mitochondrial, oxidative phosphorylation-dependent metabolic phenotype. Thus, it seems likely that a high level of ATP is required, inter alia, to maintain an elevated level of antioxidant defense.

Furthermore, differences in the effects of the 2,4-DNP and anthracyclines used simultaneously in the studied prostate cancer cell lines may be due to their different metabolic phenotype. The DU-145 and PC-3 cell lines are characterized by a stronger glycolytic phenotype compared to the LNCaP cell line, which may be the reason for the lack of a synergistic effect.

## 4. Materials and Methods

### 4.1. Cell Culture and Treatment

Three prostate cancer cell lines (PC-3, DU-145, LNCaP) were used in the study (ATCC, Manassas, VA, USA). The PC-3, DU-145 and LNCaP cells were cultured in Kaighn’s Modification of Ham’s F-12 Medium (F12-K) (ATCC, USA), Eagle’s Minimum Essential Medium (EMEM) (ATCC, USA) and in RPMI-1640 Medium (ATCC, USA), respectively. The media were supplemented with 10% FBS (fetal bovine serum) (Corning, Glendale, AZ, USA). The cells were incubated at 37 °C with 5% CO_2_ in an air atmosphere. The cells were incubated for 48 h with 100 μM 2,4-DNP and the following anthracyclines: doxorubicin (0.05–2 μM) and epirubicin (0.05–2 μM) or combined (2,4-DNP + anthracycline). The tested concentration of the 2,4-DNP was based on the previously reported studies and clinically achievable plasma concentrations as well as the observed cytotoxicity for the tested cells [9,28].

### 4.2. MTT Assay

To determine the cytotoxicity of the anthracyclines alone or combined with the 2,4-DNP on prostate cancer cell lines, an MTT assay was performed. For all the assays, the cells were seeded in the concentration of 1.5 × 10^5^ cells/mL. The tested compounds were added when 70–80% of the cell culture confluence was achieved. The MTT solution (0.5 mg/mL in phosphate-buffered saline) was added after 48 h of the cells’ incubation with the compounds. After 3 h of the cells’ incubation with the MTT solution, it was removed, and the formed formazan crystals were dissolved in DMSO. The absorbance of the obtained solutions was measured at 570 nm with the PowerWave XS microplate spectrophotometer (BioTek Instruments, Winooski, VT, USA). Each assay was conducted three times and was measured in triplicate. The IC50 values for the anthracyclines were determined using the AAT Bioquest IC50 calculator [29].

### 4.3. Assessment of Cells Morphology

For the cell morphology observation, a phase-contrast microscope, the Nikon Eclipse Ti, and NIS-Elements Imaging Software (Nikon, Tokyo, Japan) were used.

### 4.4. Cell Cycle Analysis

The cell cycle was studied with the NucleoCounter NC-3000 (ChemoMetec, Allerod, Denmark), according to the 2-Step Cell Cycle Assay protocol (ChemoMetec, Allerod, Denmark). After 48 h of incubation, the cells were detached from the plate using trypsine, suspended in 250 μL of lysis buffer (Solution 10), enriched with 10 μg/mL DAPI (4′,6-Diamidine-2′-phenylindole dihydrochloride) and incubated for 5 min at 37 °C in the dark. Then, 250 μL of the stabilization buffer (Solution 11) was added. The obtained cells’ suspension was applied onto the NC-Slide and analyzed in the NucleoCounter NC-3000. Each experiment was conducted three times with measurements in triplicate.

### 4.5. Detection of Apoptosis

An apoptosis detection was conducted with the NucleoCounter NC-3000 (ChemoMetec, Allerod, Denmark), according to the Annexin V Apoptosis Assay protocol (ChemoMetec, Allerod, Denmark). After 48 h of incubation, the cells were stained with Annexin V–FITC, Hoechst 33342 and PI, according to the manufacturer’s recommended protocol. The stained cells were applied onto the NC-Slide and analyzed in the NucleoCounter NC-3000. Each experiment was conducted three times with measurements in triplicate.

### 4.6. Determination of DNA Oxidative Damage

The LNCaP cells were seeded into six-well plates at a concentration of 1.5 × 10^5^ cells/mL. The tested compounds were added when 70–80% of the cell culture confluence was achieved. After 48 h of incubation, the cells were detached from the plate, using trypsin, and the DNA was isolated with the Syngen DNA Mini Kit (Syngen, Wroclaw, Poland), in compliance with the manufacturer’s protocol. The concentration and purity of the genomic DNA were measured with the MaestroNano micro-volume spectrophotometer (Maestrogen Inc., Hsinchu, Taiwan) and adjusted to 100 μg/mL in the TE buffer. The oxidative DNA damage was determined by measuring the number of abasic sites (AP) with the DNA Damage Quantification Kit (Dojindo, Kumamoto, Japan), according to the manufacturer’s recommended protocol. The oxidative attacks by ROS on the deoxyribose moiety led to the release of free bases from the DNA, inducing strand breaks with various sugar modifications and simple abasic sites. An aldehyde-reactive probe (ARP; N′-aminooxymethylcarbonylhydrazin-D-biotin) reacts specifically with an aldehyde group present on the open ring form of AP sites, making it possible to detect the DNA modifications that result in the formation of an aldehyde group. A biotin–avidin-specific connection and horseradish peroxidase were used for the colorimetric detection at 650 nm with the PowerWaveTM micro-plate spectrophotometer (Bio-Tek Instruments).

### 4.7. Oxidative Stress Detection

To detect the ROS in the cells, the CellROX Green Reagent (Invitrogen, Waltham, MA, USA) was used. The CellROX is a fluorogenic probe that is weakly fluorescent while in a reduced state. It exhibits bright green photostable fluorescence upon oxidation by ROS and subsequent binding to DNA, with an absorption/emission maxima of 485/520 nm. The LNCaP cells were seeded into six-well plates at a concentration of 1.5 × 10^5^ cells/mL. The tested compounds were added when 70–80% of the cell culture confluence was achieved. After 24 h of incubation, the cells were fixed with 4% PFA and subsequently stained with 5 μM CellROX Green Reagent and Hoechst 33342 (5 μg/mL) by adding them to the PBS and incubating at 37 °C for 30 min. Then, the cells were washed three times with PBS and imaged on a Nikon Eclipse Ti inverted microscope, using a 40× objective with NIS-Elements Imaging Software (Nikon, Tokyo, Japan).

### 4.8. The Level of Cellular Thiols as a Determinant of Oxidative Stress

The level of cellular thiols in the LNCaP cells was measured using the NC-3000 Vitality Assay (Chemometec, Allerod, Denmark). The principle of the method is based on the reaction of the VitaBright-48 stain with thiols and the formation of a fluorescent product. Other dyes used were as follows: acridine orange (AO: stains dead cells) and propidium iodide (PI: stains nucleated cells). The LNCaP cells were seeded into six-well plates at a concentration of 1.5 × 10^5^ cells/mL. The tested compounds were added when 70–80% of the cell culture confluence was achieved. After 24 h of incubation, the cells were harvested and stained with the above-mentioned dyes, according to the manufacturer’s recommended protocol. The stained cells were applied onto the NC-Slide and analyzed in the NucleoCounter NC-3000. Each experiment was conducted three times with measurements in triplicate.

### 4.9. Statistical Analysis

The results were presented as the mean ± SD and were analyzed with STATISTICA 13 software (StatSoft, Krakow, Poland). The values were compared using a one-way analysis of variance (ANOVA) and post hoc multiple comparisons with Tukey’s honest significant difference test (Tukey’s HSD test). The results were considered statistically significant if the *p*-value was less than 0.05.

## 5. Conclusions

New strategies for the treatment of advanced cancer diseases, including prostate cancer, are constantly being searched for. One of them may be the sensitization of neoplastic cells with a known metabolic phenotype, with the substances disrupting the various pathways of energy metabolism. The synergistic effect of the 2,4-DNP and anthracyclines on the LNCaP prostate cancer cells with an oxidative phenotype, revealed in this study, may be the starting point for further research, including other types of cancers and compounds.

## Data Availability

The data presented in this study are available on request from the corresponding author.
